# Formation and preliminary characterization of mercury-reactive immunoglobulin Y antibodies in laying hens using HgCl_2_–protein hapten conjugates

**DOI:** 10.14202/vetworld.2026.2597-2605

**Published:** 2026-06-25

**Authors:** Wayan Wariata, Made Sriasih, Anwar Rosyidi, Sulaiman Ngongu Depamede

**Affiliations:** Laboratory of Biotechnology and Animal Products, Faculty of Animal Science, University of Mataram, West Nusa Tenggara, Indonesia

**Keywords:** avian antibodies, environmental Hg detection, hapten–carrier conjugate, immunoglobulin Y, laying hens, Hg IgY, Hg-reactive antibodies, veterinary toxicology

## Abstract

**Background and Aim:**

Mercury (Hg) is a persistent environmental toxin that poses significant risks to animal and human health through contaminated feed, water, and ecosystems. Conventional instrumental methods for Hg detection are laboratory-intensive, while immunochemical approaches require highly specific antibodies. Hg ions are poorly immunogenic and therefore require hapten–carrier conjugation. This proof-of-concept study aimed to evaluate the feasibility of generating functional Hg-reactive immunoglobulin Y (IgY) antibodies in laying hens using Hg chloride (HgCl_2_)–protein conjugates, offering an avian alternative to mammalian antibody production for veterinary toxicology and environmental monitoring applications.

**Materials and Methods:**

HgCl_2_ was conjugated to keyhole limpet hemocyanin (KLH) using 3-maleimidobenzoyl-N-hydroxysuccinimide ester (MBS) as crosslinker and emulsified with Freund’s adjuvants. Two Hy-Line Brown laying hens received subcutaneous immunizations (primary dose: 100 μg antigen in complete Freund’s adjuvant [CFA]; booster doses: 50 μg antigen in incomplete Freund’s adjuvant on days 14 and 28). Due to irregular egg production, serum was used as the antibody source. Antibody reactivity was assessed by indirect enzyme-linked immunosorbent assay (ELISA) using HgCl_2_–bovine serum albumin (BSA) as coating antigen. IgY was purified by caprylic acid and ammonium sulfate precipitation and characterized by sodium dodecyl sulfate–polyacrylamide gel electrophoresis (SDS-PAGE). The study was conducted under strict ethical approval (No. 2403/FapetUN/ETIK/2024) and biosafety protocols, with a minimal cohort size justified by constraints on Hg handling.

**Results:**

In the single hen that completed the full immunization protocol, post-immunization serum (day 42) showed strong reactivity at 10 μg/mL HgCl_2_–BSA coating. Optical density at 450 nm (OD_450_) ranged from 0.42 to 0.67 compared with pre-immune baseline values below 0.22, producing a fold increase over pre-immune serum of 1.93–11.41× (9 of 15 conditions >5×; 6 of 15 conditions >8×). Intra-assay coefficient of variation remained below 10% (mean 4.27%). Purified IgY displayed intact heavy chain (~65 kDa) and light chain (~25 kDa) bands on reducing SDS-PAGE, confirming successful enrichment.

**Conclusion:**

This preliminary observation demonstrates successful induction of Hg-reactive IgY antibodies in a laying hen using a hapten–carrier immunization strategy. The robust ELISA discrimination supports further development of IgY-based tools for Hg screening. Future studies must include ≥3 biological replicates, hapten–density quantification, specificity controls, and competitive assay formats for proper validation.

## INTRODUCTION

Mercury (Hg) is a persistent environmental contaminant with recognized neurotoxic and nephrotoxic effects in animals and humans [1]. In veterinary contexts, Hg exposure may occur through contaminated aquatic ecosystems, fish-derived feed ingredients, or environmental accumulation in agricultural settings [2, 3]. Surveillance strategies that enable screening of feed, water, or biological matrixes are therefore relevant to animal health and food safety assurance. Instrument-based methods such as atomic absorption spectroscopy and inductively coupled plasma mass spectrometry provide high analytical sensitivity but require centralized laboratory infrastructure [4–6]. Immunochemical detection offers an alternative based on molecular recognition, enabling potential development of portable or field-adaptable formats [7, 8]. However, Hg ions lack intrinsic immunogenicity and must be conjugated to carrier proteins to induce antibody formation. The unique coordination chemistry of inorganic Hg and its direct cytotoxicity pose additional challenges for hapten design, because both linker chemistry and hapten–density critically affect immunogenicity and enzyme-linked immunosorbent assay (ELISA) signal intensity.

Although previous studies have demonstrated Hg-specific immunoglobulin (Ig)G production in mammalian systems [9, 10], avian IgY platforms remain largely underexplored for inorganic metal haptens such as Hg chloride (HgCl_2_). Existing mammalian monoclonal antibodies against Hg-chelate complexes have shown promise in laboratory-based assays; however, they are associated with several limitations, including high production costs, ethical concerns related to mammalian serum collection, and potential interference from mammalian Fc receptor interactions in various diagnostic matrixes. In contrast, IgY antibodies offer distinct advantages, such as phylogenetic distance from mammalian systems, which can yield higher specificity; minimal interaction with mammalian complement and Fc receptors; and the possibility of non-invasive harvesting from egg yolk when laying is consistent. Despite these potential benefits, no published reports have successfully demonstrated the feasibility of generating functional Hg-reactive IgY antibodies in laying hens using HgCl_2_–protein hapten conjugates. Critical aspects, including optimal hapten–density, suitable carrier protein selection, linker chemistry effects, and specificity profiles against inorganic Hg, remain completely uncharacterized in avian systems. This gap limits the development of cost-effective, ethically favorable, and scalable immunochemical tools for Hg detection in veterinary toxicology and environmental monitoring, particularly in resource-limited settings where mammalian antibody production may be less practical.

Establishing that Hg-reactive IgY can be produced is therefore of significance for veterinary diagnostics, food safety, and environmental monitoring platforms. IgY offers advantages including species-specific antibody production, reduced interaction with mammalian Fc receptors, and alignment with animal welfare principles [11–15]. Furthermore, egg yolk is a non-invasive and scalable source of IgY when egg production is regular, offering an additional practical advantage over serum-based mammalian antibody systems.

This proof-of-concept study aimed to evaluate the feasibility of producing Hg-reactive IgY antibodies in laying hens immunized with HgCl_2_–protein conjugates, while adhering to strict biosafety and ethical constraints. Specifically, the study sought to determine whether HgCl_2_–keyhole limpet hemocyanin (KLH) conjugates formulated with Freund’s adjuvants could elicit a detectable antigen-specific IgY response measurable by indirect ELISA using HgCl_2_–bovine serum albumin (BSA) as the coating antigen. The study was deliberately designed as a binary feasibility assessment (presence or absence of a detectable response) rather than a fully powered experiment intended to estimate population parameters or detailed assay performance metrics. Secondary objectives included preliminary purification of the induced IgY and basic structural characterization by sodium dodecyl sulfate–polyacrylamide gel electrophoresis (SDS-PAGE). Findings are reported as a preliminary technical observation to provide a well-documented starting point, including detailed immunization and assay protocols, for future adequately powered studies with sufficient biological replication.

## MATERIALS AND METHODS

### Ethical approval

This study was approved by the Research Ethics Committee of the Faculty of Animal Science, University of Mataram (Approval No. 2403/FapetUN/ETIK/2024, dated before commencement of the study). All procedures involving live animals were performed in strict accordance with the approved protocol and complied with the Indonesian Law No. 41 of 2014 concerning Animal Protection and Welfare, as well as the institutional guidelines for the care and use of laboratory animals. The experimental design adhered to the principles of the 3Rs (Replacement, Reduction, and Refinement) to minimize animal use and suffering. A minimal cohort of two laying hens was justified based on biosafety constraints associated with Hg handling and the study’s proof-of-concept nature. Daily monitoring of feed intake, behavior, body weight, and general health status was conducted throughout the experimental period. Humane endpoints were predefined, and any animal showing clinical signs of distress was to be immediately removed from the study and provided appropriate veterinary care or euthanized humanely. Hg handling and waste disposal complied with institutional biosafety-level protocols and hazardous-materials regulations. No *in vivo* procedures beyond subcutaneous immunization and routine blood sampling from the wing vein were performed. The study was conducted in accordance with Animal Research: Reporting of *In Vivo* Experiments (ARRIVE) 2.0 guidelines for transparent reporting of animal research.

### Study period and location

The study was conducted between October and December 2024 at the Laboratory of Biotechnology and Animal Products, Faculty of Animal Science, University of Mataram, West Nusa Tenggara, Indonesia.

### Study design

This was a preliminary proof-of-concept immunization study using a minimal cohort of two laying hens. The design focused exclusively on determining the feasibility of inducing a detectable IgY response rather than on statistical inference or population-level estimates.

### Sample size rationale

A minimal cohort of two hens was selected a priori based on biosafety and ethical considerations specific to Hg handling, which impose strict limits on animal numbers under institutional protocols. This sample size does not provide sufficient statistical power for inferential analysis and was not intended to. The study was designed exclusively to determine whether the immunization protocol could elicit a detectable IgY response, a binary feasibility question, rather than to estimate population parameters or assay performance metrics.

### Biosafety and monitoring

All Hg handling procedures were conducted inside a certified chemical safety cabinet using appropriate personal protective equipment. Hg-containing waste was disposed of in accordance with institutional hazardous waste protocols. Birds were monitored daily for feed intake, behavior, and general health status. Body weight was recorded weekly. Egg production was noted throughout the immunization period. No clinical signs consistent with Hg intoxication were observed.

### Preparation of HgCl_2_–protein conjugates

HgCl_2_ (Merck, Darmstadt, Germany) was conjugated to KLH (Sigma-Aldrich, St. Louis, MO, USA) using 3-maleimidobenzoyl-N-hydroxysuccinimide ester (Sigma-Aldrich) as a crosslinker, with minor modifications to established protocols [9, 16]. Briefly, 3-maleimidobenzoyl-N-hydroxysuccinimide ester (2 mg, dissolved in dimethylformamide) was added to KLH (5 mg) in 0.1 M phosphate buffer (pH 7.2) and incubated at room temperature for 60 min with gentle agitation. Excess 3-maleimidobenzoyl-N-hydroxysuccinimide ester was removed by gel filtration using a Sephadex G25 column equilibrated with phosphate-buffered saline (PBS; pH 7.2). Protein-containing fractions were identified qualitatively using Bradford reagent and pooled. HgCl_2_ (molar ratio approximately 50:1, Hg:KLH) was then added to the maleimide-activated KLH and incubated at room temperature for 3 h. The resulting conjugate was dialyzed against PBS (pH 7.4) overnight at 4°C. HgCl_2_–BSA (Sigma-Aldrich) was prepared in parallel using the same molar ratio for ELISA coating to minimize carrier-specific binding. Formal characterization of conjugation efficiency, including hapten–density quantification (e.g., by trinitrobenzene-sulfonic acid assay or matrix-assisted laser desorption/ionization time-of-flight mass spectrometry) and spectroscopic confirmation of Hg incorporation into either the KLH or BSA conjugates, was not performed in this proof-of-concept study. This is acknowledged as a limitation. However, in early-phase hapten immunology studies where the primary objective is to determine whether an immune response can be elicited, the functional outcome of the immunization, specifically, the detection of antigen-reactive antibodies by indirect ELISA, serves as indirect evidence that conjugation was sufficient to produce an immunogenic product [9, 17–19]. Formal characterization of the conjugate, including hapten–density determination and confirmation of successful Hg incorporation into both KLH and BSA conjugates, is planned as a priority step in the next phase of this research.

### Animals and immunization

Two healthy laying hens (commercial Hy-Line Brown), aged approximately 28–30 weeks, were sourced from a local commercial flock and acclimatized for 14 days prior to immunization. Hens were housed in individual cages under standard conditions (12 h light/dark cycle, 27°C, ad libitum commercial layer feed and water). Each hen received a subcutaneous injection of HgCl_2_–KLH at three to four sites on the back and neck region. The injection volume per site was 0.25 mL (total 0.75–1.0 mL per hen). The primary dose was equivalent to 100 μg KLH per animal; second and third booster doses were 50 μg per animal. The primary immunization (Day 0) used complete Freund’s adjuvant (CFA; Sigma-Aldrich) emulsified 1:1 (v/v) with antigen solution. Booster immunizations at Day 14 and Day 28 used incomplete Freund’s adjuvant (Sigma-Aldrich) emulsified 1:1 (v/v). Emulsification was performed on ice until a stable water-in-oil emulsion was confirmed by the drop test. One hen (initial body weight 1,825 g) died on 16 November 2024, approximately three weeks after the second booster immunization. No clinical signs consistent with Hg intoxication were observed prior to death. No necropsy was performed; the carcass was disposed of by burial in accordance with institutional biosafety procedures. Serum from this animal, collected prior to death, was not included in final analyses due to incomplete post-immunization sampling. The surviving hen (body weight 1,845 g) completed the full immunization protocol and provided all samples used in subsequent analyses.

### Serum collection

Blood samples (3 mL per collection) were collected from the wing vein prior to immunization (pre-immune) and biweekly thereafter. IgY extraction from egg yolk was not performed in this study because both hens exhibited irregular egg-laying patterns throughout the immunization period, which precluded consistent yolk collection. Serum was therefore used as the sole antibody source [20]. Based on screening results, serum obtained at Day 42 (two weeks after the third immunization) was selected for comparative analysis.

### Indirect ELISA

ELISA plates were coated overnight at 4°C with HgCl_2_–BSA at three concentrations (20, 10, or 5 μg/mL; 100 μL/well in PBS). Following blocking with 3% skim milk in PBST (0.05% Tween-20 in PBS) for 1 h at 37°C, serum samples (50 μL/well at five serial concentrations: 20, 10, 5, 2.5, and 1.25 μg/mL total protein) were applied in triplicate wells and incubated for 1 h at 37°C. Horseradish peroxidase-conjugated goat anti-chicken IgY (Invitrogen, Carlsbad, CA, USA) at 1:20,000 dilution was used as secondary antibody, followed by 3,3’,5,5’-Tetramethyl-benzidine substrate development (10 min, room temperature) and absorbance measurement at 450 nm. The coating concentration of 10 μg/mL was selected for primary reporting because it yielded the highest fold increase over pre-immune serum and an acceptable background across the three antigen concentrations tested during checkerboard optimization. A formal endpoint titer was not calculated in this study; antibody reactivity was instead characterized by fold-change in OD_450_ relative to pre-immune baseline and by fold increase over pre-immune serum across the serum concentration range tested, which are appropriate descriptive metrics for a single-replicate preliminary study [21]. Pre-immune serum from the same individual hen served as the negative control. No carrier-only or cross-reactivity controls with other metal ions were included in this proof-of-concept study; these are identified as limitations and planned for future experiments.

### IgY purification and SDS-PAGE

High-titer serum was purified using caprylic acid precipitation followed by ammonium sulfate fractionation [22]. Briefly, serum was diluted 1:4 in 0.1 M sodium acetate buffer (pH 4.8). Caprylic acid (25 μL per mL diluted serum) was added dropwise under stirring, and the mixture was centrifuged at 10,000 × *g* for 30 min. The supernatant was filtered, ammonium sulfate was added to 45% saturation, the mixture was gently agitated for 30 min at 0–4°C, and then centrifuged at 10,000 × *g* for 30 min. The precipitate was collected, resuspended in PBS, and dialyzed against PBS overnight at 4°C. Protein concentration was determined by A280 spectrophotometry. Purified IgY was analyzed by 10% SDS-PAGE under reducing conditions. Loading volume was standardized to 10 μg protein per lane. Molecular weight markers (BLUelf Prestained Protein Ladder, GeneDireX, Inc., BN Korea) were included in all gels.

### Statistical analysis

ELISA results are presented as mean ± standard deviation of triplicate wells (technical replicates; n = 3 well replicates per condition from a single biological replicate). Intra-assay coefficient of variation (CV%) was maintained below 10% across all 15 conditions tested (range: 0.38–7.68%; mean: 4.27%). The fold increase over pre-immune serum was calculated as post-immunization OD divided by corresponding pre-immunization OD at identical serum concentrations. Given the single biological replicate, statistical inference was not performed; effect magnitude and fold-change were interpreted descriptively [23–25],

## RESULTS AND DISCUSSION

**Figure 1:**
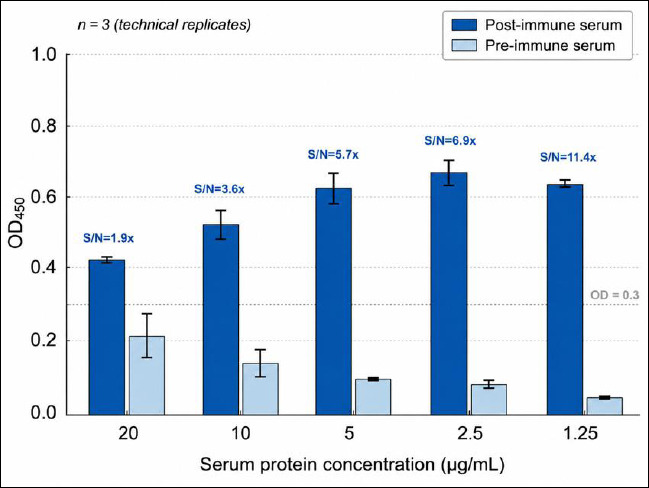
Enzyme-linked immunosorbent assay (ELISA) reactivity of anti-mercury IgY from a single biological replicate. Coating antigen: HgCl_2_–BSA at 10 μg/mL (selected based on the highest fold increase over pre-immune serum across three concentrations tested: 5, 10, and 20 μg/mL). Serum protein concentrations applied in descending order: 20, 10, 5, 2.5, and 1.25 μg/mL. Post-immune serum: collected at Day 42 (two weeks after third immunization); OD_450_ range 0.42–0.67. Pre-immune serum: collected at Day 0 prior to immunization; OD_450_ range 0.056–0.218. Fold increase over pre-immune serum range at this coating concentration: 1.93–11.41×. n = 3 refers to technical replicates (triplicate wells) from one individual hen; it does not represent independent biological replicates. Error bars represent standard deviation (intra-assay CV range: 0.38–7.68%). The decrease in post-immune OD at higher serum concentrations is consistent with a high-dose hook effect in indirect ELISA formats and does not indicate absent reactivity.

In the single hen that completed the full immunization protocol, at the selected coating concentration of 10 μg/mL HgCl_2_–BSA, post-immunization serum OD^450^ values ranged from 0.42 to 0.67, compared with pre-immune baseline values below 0.22, representing a 1.93 to 11.41-fold increase (Figure 1). This is consistent with, though not sufficient on its own to confirm, typical seroconversion profiles reported for avian IgY generated against hapten–carrier conjugates. Figure 1 presents data for the 10 μg/mL HgCl_2_–BSA coating, selected because it yielded the most discriminative fold increase over pre-immune serum during checkerboard optimization across the three antigen concentrations tested (5, 10, and 20 μg/mL). The x-axis represents serum protein concentrations in descending order (20, 10, 5, 2.5, and 1.25 μg/mL), consistent with the standard serial-dilution presentation.

The pattern of decreasing post-immune OD at higher serum concentrations is consistent with a high-dose hook effect commonly observed in indirect ELISA formats and does not indicate absent reactivity. The n = 3 noted in the figure legend refers exclusively to technical replicates (triplicate wells) from this single biological replicate, not to independent animals. Studies on small-molecule immunogens demonstrate that antibody titers and ELISA absorbance are highly dependent on hapten–density, linker chemistry, and carrier protein selection [17–19, 26], parameters that should be systematically characterized in future work.

The fold increase over pre-immune serum, calculated as the ratio of post-immune to pre-immune OD_450_ at each serum concentration, ranged from 1.93 to 11.41 at the 10 μg/mL coating concentration, and from 1.93 to 11.41 across all three coating concentrations combined. Nine of 15 conditions exceeded an S/N of 5×, and 6 of 15 conditions exceeded 8×, consistent with strong preliminary discrimination between immune and baseline sera. The S/N values observed in this single-replicate experiment are indicative of, but not sufficient on their own to confirm, analytical discrimination. In immunoassay development, S/N values above 5 are widely considered acceptable for preliminary feasibility assessment, whereas higher thresholds (≥8–10) are more typical in validated diagnostic ELISAs [23–25]. However, because these values derive from a single individual hen, they carry unknown biological uncertainty and should not be interpreted as representative of the species, strain, or protocol performance in general. Further enhancement may be achieved through affinity purification, which often improves signal amplitude and reduces background [27, 28].

### SDS-PAGE analysis of purified IgY

**Figure 2:**
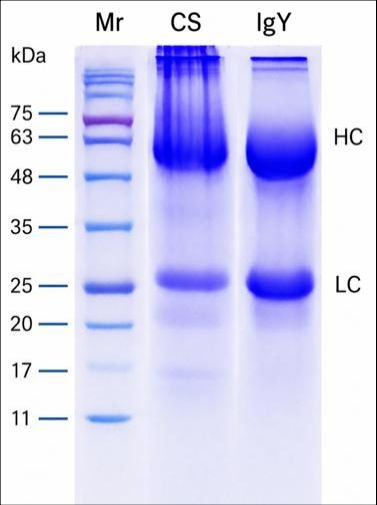
Sodium dodecyl sulfate–polyacrylamide gel electrophoresis profile of IgY purification under reducing conditions (10% resolving gel). Lane 1, Mr: BLUelf Prestained Protein Ladder; Lane 2, CS: crude serum (total serum protein before purification); Lane 3, IgY: purified IgY fraction after caprylic acid and 45% ammonium sulfate precipitation (10 μg loaded). HC: heavy chain (~65 kDa); LC: light chain (~25 kDa). The reduction in background protein bands between CS and IgY lanes confirms enrichment of IgY during purification. A minor band at approximately 48 kDa in the IgY lane may represent a co-purified serum protein or IgY degradation fragment and does not affect the interpretation of the dominant HC and LC bands.

SDS-PAGE analysis (Figure 2) demonstrates the progressive enrichment of IgY through the purification process. The CS shows a complex mixture of proteins across a wide molecular weight range, reflecting the heterogeneous composition of whole serum. In contrast, the purified IgY lane shows two dominant bands: an HC at approximately 65 kDa and an LC at approximately 25 kDa, consistent with canonical IgY architecture [28–30]. The marked reduction in background protein bands between lane 2 (CS) and lane 3 (purified IgY) provides visual confirmation that the caprylic acid and ammonium sulfate precipitation steps were effective in enriching IgY from the serum matrix. A minor band at approximately 48 kDa is visible in the purified IgY lane. This band may represent a co-purified serum protein, an IgY degradation fragment, or a partially reduced IgY subunit. Its presence does not affect the interpretation of the dominant HC and LC bands, but underscores the need for additional purification steps, such as affinity chromatography, in future work to achieve higher purity. IgY differs structurally from mammalian IgG by lacking a hinge region and exhibiting altered Fc composition, reducing interactions with mammalian Fc receptors and complement components [13, 15]. This property can enhance assay specificity by minimizing Fc-mediated non-specific binding in mammalian matrixes [15, 31–33]. The overall band profile supports the structural integrity of the purified material; however, functional binding specificity to Hg, as opposed to the carrier protein or MBS linker, was not confirmed in this study and represents a key gap to be addressed by Western blot or competitive inhibition assays in future work [34].

### Limitations of the study

The primary limitation of this study is the single biological replicate available for full analysis. This precludes estimation of inter-individual variability, biological CV, or population-level antibody titers. It also means that the ELISA performance metrics reported here, fold-change and fold increase over pre-immune serum, reflect the response of one individual hen and may not be representative of the species or strain. Without multiple replicates, statistical tests cannot be applied, confidence intervals cannot be reported, and effect size estimates, while descriptively large, carry unknown uncertainty. Additionally, a formal characterization of conjugation efficiency, including hapten–density for both HgCl_2_–KLH and HgCl_2_–BSA conjugates, was not performed. The absence of this data means that the exact immunogenic load delivered cannot be confirmed retrospectively, and it is not possible to rule out that the observed ELISA signal reflects reactivity to the MBS linker or carrier protein rather than to Hg specifically. Nevertheless, the functional ELISA response observed, a 1.93 to 11.41-fold increase in OD_450_ and fold increase over pre-immune serum reaching 11.41×, provides indirect evidence that the conjugation procedure produced a product capable of stimulating antibody formation, which represents the minimum requirement for a proof-of-concept feasibility assessment [9, 10, 19]. The absence of carrier-specificity controls and cross-reactivity panels with other metal ions further limits confidence in the Hg-hapten-directed nature of the observed signal. These constraints are acknowledged in accordance with the Animal Research: Reporting of In Vivo Experiments 2.0 reporting standards [35] and serve as the primary justification for positioning this work as a preliminary technical report rather than an experimental study with validated outcomes.

Notwithstanding these limitations, the magnitude of the observed effect in this individual animal is encouraging and sufficient to justify the next phase of investigation. Future studies incorporating ≥3 biological replicates, standardized effect sizes (e.g., Cohen’s d), hapten–density characterization, and specificity controls would substantially strengthen the evidence base [36].

## CONCLUSION

This preliminary proof-of-concept study successfully demonstrated the induction of Hg-reactive IgY antibodies in a laying hen immunized with HgCl_2_–KLH conjugates. In the single hen that completed the full immunization protocol, post-immunization serum showed clear reactivity by indirect ELISA, with OD_450_ values ranging from 0.42 to 0.67 at 10 μg/mL HgCl_2_–BSA coating (compared with pre-immune values <0.22), yielding fold increase over pre-immune serum of 1.93–11.41× (9 of 15 conditions >5× and 6 of 15 conditions >8×). Purified IgY exhibited intact heavy (~65 kDa) and light (~25 kDa) chains on reducing SDS-PAGE, confirming successful enrichment.

These findings open the possibility of developing avian IgY-based immunochemical tools for Hg screening in animal feed, water, and biological matrixes. Such tools could offer cost-effective, ethical, and field-applicable alternatives to traditional instrumental methods and mammalian antibody systems in veterinary toxicology and environmental monitoring.

The study employed a well-defined hapten–carrier conjugation strategy with Freund’s adjuvants and validated antibody reactivity using quantitative indirect ELISA under optimized conditions, while maintaining strict biosafety protocols for Hg handling.

The results are derived from a single biological replicate due to biosafety constraints and the death of one hen, precluding statistical inference, assessment of inter-individual variability, and full validation of assay performance. Additionally, hapten–density quantification, carrier-specificity controls, and cross-reactivity testing with other metal ions were not performed.

Future studies should include ≥3 biological replicates, formal hapten–density characterization, specificity controls, competitive ELISA formats, and consistent egg yolk IgY extraction when regular laying is achieved. Affinity purification and advanced validation will further strengthen the platform for practical diagnostic applications.

In conclusion, this work provides encouraging preliminary evidence that functional Hg-reactive IgY can be generated in laying hens and establishes a documented foundation with detailed protocols for subsequent research. With adequate replication and refinement, IgY-based approaches hold strong potential as an ethical and scalable alternative for Hg detection in veterinary and environmental contexts.

## DATA AVAILABILITY

The supplementary data can be made available from the corresponding author upon request.

## AUTHORS’ CONTRIBUTIONS

WW: Designed the study, managed data collection, established the research framework, and prepared the initial manuscript draft. MS: Conducted the data analysis and created the visualization, contributed to reviewing and revising the manuscript. AR: Conducted the data analysis and created the visualization, contributed to reviewing and revising the manuscript. SND: Designed the study, managed data collection, established the research framework, and prepared the initial manuscript draft. All authors have read and approved the final manuscript.
